# Involvement of the SAGA and TFIID coactivator complexes in transcriptional dysregulation caused by the separation of core and tail Mediator modules

**DOI:** 10.1093/g3journal/jkac290

**Published:** 2022-11-04

**Authors:** Moustafa M Saleh, Heather A Hundley, Gabriel E Zentner

**Affiliations:** Department of Biology, Indiana University, Bloomington, IN 47405, USA; Department of Biology, Indiana University, Bloomington, IN 47405, USA; Department of Biology, Indiana University, Bloomington, IN 47405, USA

**Keywords:** Mediator, SAGA, TFIID, nsRNA-seq, ChEC-seq

## Abstract

Regulation of RNA polymerase II transcription requires the concerted efforts of several multisubunit coactivator complexes, which interact with the RNA polymerase II preinitiation complex to stimulate transcription. We previously showed that separation of the Mediator core from Mediator’s tail module results in modest overactivation of genes annotated as highly dependent on TFIID for expression. However, it is unclear if other coactivators are involved in this phenomenon. Here, we show that the overactivation of certain genes by Mediator core/tail separation is blunted by disruption of the Spt-Ada-Gcn5-Acetyl transferase complex through the removal of its structural Spt20 subunit, though this downregulation does not appear to completely depend on reduced Spt-Ada-Gcn5-Acetyl transferase association with the genome. Consistent with the enrichment of TFIID-dependent genes among genes overactivated by Mediator core/tail separation, depletion of the essential TFIID subunit Taf13 suppressed the overactivation of these genes when Med16 was simultaneously removed. As with Spt-Ada-Gcn5-Acetyl transferase, this effect did not appear to be fully dependent on the reduced genomic association of TFIID. Given that the observed changes in gene expression could not be clearly linked to alterations in Spt-Ada-Gcn5-Acetyl transferase or TFIID occupancy, our data may suggest that the Mediator core/tail connection is important for the modulation of Spt-Ada-Gcn5-Acetyl transferase and/or TFIID conformation and/or function at target genes.

## Introduction

Regulation of RNA polymerase II (RNAPII) transcription is an intricately regulated process that must simultaneously balance the cell’s requirements for housekeeping gene expression and the need for gene expression to be responsive to external stimuli. Multisubunit coactivator complexes are key regulators of both constitutive and induced gene expression. Coactivators exert their influence on RNAPII transcription through a number of means, from modification of nucleosomes at various *cis*-regulatory elements to modulating the shift of RNAPII from initiation to elongation (Thomas and Chiang 2006; Krasnov *et al.* 2016; Cramer 2019). Perhaps the best-known coactivator complexes are Mediator, TFIID, and Spt-Ada-Gcn5-Acetyl transferase (SAGA) (Thomas and Chiang 2006; Schier and Taatjes 2020). These coactivators co-occupy the promoters and enhancers of almost all genes (Jeronimo and Robert 2014; Grünberg *et al.* 2016; Baptista *et al.* 2017; Donczew *et al.* 2020). However, how these coactivators influence each other’s activities and subsequently affect RNAPII transcription is not well understood.

SAGA, TFIID, and Mediator have diverse functions. One of the best-understood coactivator functions is TBP delivery to promoters by SAGA and TFIID (Timmers 2021). TBP delivered to promoters nucleates the assembly of the preinitiation complex (PIC), of which both Mediator and TFIID are components *in vivo* (Cramer 2019)*.* Mediator is a multisubunit complex composed of 25 subunits in yeast arranged into multiple structural modules: head, middle, tail and the transiently associated kinase module (Soutourina 2018). Functionally, Mediator can be divided into core Mediator (cMed), which is composed of the head and middle modules held together by the scaffold subunit Med14 and the tail module, which interacts with transcription factors (TFs) (Brzovic *et al.* 2011; Cevher *et al.* 2014; Plaschka *et al.* 2015). Mediator functional modules have differential effects on gene expression. These effects are associated with different features of gene regulation such as promoter architecture and dependence on other coactivator complexes (Van De Peppel *et al.* 2005; Ansari *et al.* 2012; Tourigny *et al.* 2021; Saleh *et al.* 2021).

Previous studies have demonstrated that Mediator can also influence the recruitment of SAGA to specific genes (Qiu *et al.* 2005; Yarrington *et al.* 2020). SAGA is composed of 19 subunits arranged into 4 distinct functional modules: a histone acetyl transferase module, a deubiquitylase module, an activator binding module (Tra1), and a core module that holds all of the different functional modules together and directly interacts with TBP (Papai *et al.* 2020; Wang *et al.* 2020; Cheon *et al.* 2020). The Mediator tail module and SAGA regulate an overlapping set of genes (Ansari *et al.* 2012), the promoters of which tend to contain TATA boxes, which are high-affinity binding sites for TBP (Huisinga and Pugh 2004).

Conversely, transcription from promoters without consensus TATA boxes is largely unaffected by mutations in SAGA and Mediator tail subunits (Huisinga and Pugh 2004; Ansari *et al.* 2012), and these promoters generally show high occupancy of TFIID subunits (Rhee and Pugh 2012). In budding yeast, TFIID is composed of TBP and 14 TBP-associated factors (Taf1-14) (Sanders *et al.* 2002; Tora 2002). These subunits are arranged into 3 lobes: lobe A (Taf1 lobe), lobe B (Twin lobe), and lobe C (Taf2 lobe) (Kolesnikova *et al.* 2018). Some TAFs of TFIID are shared with other complexes: Taf5, Taf6, Taf9, Taf10, and Taf12 are shared with SAGA (Grant *et al.* 1998; Han *et al.* 2014), while Taf14 is shared with 4 other complexes with various functions related to transcription (Henry *et al.* 1994; Cairns *et al.* 1996; Peterson *et al.* 1998; John *et al.* 2000). The TFIID-specific Tafs Taf1, Taf11 and Taf13 contact TBP in TFIID (Anandapadamanaban *et al.* 2013; Gupta *et al.* 2017; Kolesnikova *et al.* 2018). TFIID binds several regulatory sequences alongside Mediator and SAGA (Rhee and Pugh 2012; Grünberg *et al.* 2016; Baptista *et al.* 2017; Warfield *et al.* 2017), and, like Mediator and SAGA, is generally required for RNAPII transcription from all genes (Baptista *et al.* 2017; Warfield *et al.* 2017; Tourigny *et al.* 2021).

In this study, we investigate how SAGA and TFIID influence the transcriptional dysregulation induced by cMed/tail separation via removal of the connecting Med16 subunit. Our results indicate that Med16 depletion has a minimal effect on SAGA recruitment to Med16-regulated genes. Furthermore, disrupting SAGA by depleting the core subunit Spt20 has effects reminiscent of cMed/tail separation on the nascent transcriptome and partially mitigates Med16-depletion-dependent transcriptional overactivation. As anticipated, abrogation of TFIID function by depletion of Taf13 robustly eliminates cMed/tail separation-dependent transcriptional overactivation. The observed transcriptional changes do not appear to be strictly attributable to alterations in SAGA or TFIID association with the genome, suggesting that Mediator structural integrity may play a role in conformation and/or function of these coactivators when bound to target gene regulatory sequences.

## Materials and methods

### Yeast methods

*S*accharomyces *cerevisiae* cells were cultured in Yeast extract, peptone and dextrose (YPD) media at 30°C. SAGA subunits, Spt3 and Spt8, were tagged with 3xFLAG-MNase using pGZ110 (*TRP1* marker). SBY13674 (W303 expressing *pGPD1-OsTIR1-LEU2*, kindly provided by Sue Biggins) was used as the background to generate all AID strains. Taf13 and Spt20 were tagged with 3xHA-IAA7 using pGZ360 (HIS3MX6 marker), while Med16 was tagged with 3xV5-IAA7 using pL260/pSB2065 (kanMX6 marker). Supplementary Table 1 contains the complete genotype of all strains used in this study.

### ChEC-seq

All ChEC-seq experiments except for Spt3 ChEC-seq in Spt20-AID were done as previously described (Tourigny *et al.* 2018). Briefly, cells were grown to mid-log phase in YPD and then cells were pelleted, washed with buffer A thrice after which the cell pellet was resuspended in buffer A supplemented with digitonin (final concentration of 0.1%) to permeabilize the cells. Following, CaCl_2_ was added to the cell suspension (final concentration of ∼2 mM) and then incubated at 30°C for 1 min before the suspension was transferred to another tube containing 100 µl of stop buffer. For the Spt3 ChEC-seq in Spt20-AID, ChEC protocol was similar to the ChEC protocol described with the following differences: (1) CaCl_2_ was added to the digitonin-permeabilized cell suspension at a final concentration of ∼0.2 mM and (2) the CaCl_2_ supplemented cell suspension was incubated for 5 min at 30°C. The rest of the protocol includes DNA extraction, RNase treatment, and size selection, which were done as previously described (Tourigny *et al.* 2018). ChEC-seq libraries were prepared by the Indiana University Center for Genomics and Bioinformatics (CGB) using the NEBNext Ultra II DNA Library Prep Kit for Illumina. Libraries were sequenced for 38 or 75 cycles in paired-end mode on the Illumina NextSeq 500 platform at the CGB.

### nsRNA-seq

nsRNA was done as previously described (Saleh *et al.* 2021). Briefly, cells grown in YPD were split into 2 equal fractions treated with either 3-IAA (final concentration of 0.5 mM) or an equivalent volume of DMSO for 30 min. Following, cultures were treated with 4-thiouracil (4tU) (final concentration of 5 mM) for 6 min at 30°C. Then, cells were pelleted and washed with ice-cold PBS twice before centrifugation and cell pellets were kept on ice. The separately labeled spike-in *S. pombe* culture was combined with the *S. cerevisiae* 4tU-labeled cells to a final ratio of 1:4 (*S. pombe* to budding yeast) based on the optical density of the cultures at 600 nm. RNA extraction, biotinylation, pull-down, and final purification were done as described (Saleh *et al.* 2021). rRNA was depleted from nsRNA using Terminator 5′-Phosphate-Dependent Exonuclease (Lucigen TER51020) digestion as per the manufacturer’s protocol, and rRNA-depleted nascent RNA was purified and concentrated using RNAClean XP clean beads (1.8:1 beads: sample ratio). Libraries were prepared by the CGB using the TruSeq Stranded Total RNA kit for Illumina and sequenced as described for ChEC-seq.

### Data analysis

#### nsRNA-seq

nsRNA-seq data were analyzed as described previously (Saleh *et al.* 2021). Briefly, STAR (2.6.1a) was used to align reads and generate counts per gene for both sacCer3 (budding yeast) and ASM294 (fission yeast) genomes (Dobin *et al.* 2013). The R package DESeq2 was used for spike-in normalization and differential expression analysis (Love *et al.* 2014, p. 2). Data tables generated from differential gene expression analysis by DESeq2 are in Supplementary Table 2. The coactivator-redundant (CR) and TFIID gene categorization used was from the Hahn lab (Donczew *et al.* 2020). Statistical tests were done using base R.

#### ChEC-seq

Reads were aligned to the sacCer3 genome build using Bowtie2 (version 2.3.2) (Langmead and Salzberg 2012, p. 2) with the settings previously described (Saleh *et al.* 2021). Tag directories were made from SAM files with HOMER (Heinz *et al.* 2010) using default settings. BAM files were generated from SAM files using SAMtools (v1.9) (Li *et al.* 2009). Bigwigs and heatmaps and PCA plots were generated using DeepTools (v3.4.1) (Ramírez *et al.* 2016) using the settings and commands previously described for Bigwigs (Saleh *et al.* 2021). Using HOMER *annotatepeaks.pl*, total normalized ChEC-seq signal in 2-kb windows centered on the TSSs of all genes in the sacCer3 annotation (6,672 genes) was determined. These normalized counts were then used to generate Spearman correlation plots using the R package “corrplot”. ChEC-seq internal normalization boxplots were generated as previously described (Saleh *et al.* 2021).

## Results

### cMed/tail separation reduces Spt8 occupancy at Med16-down genes

**Fig. 1.**
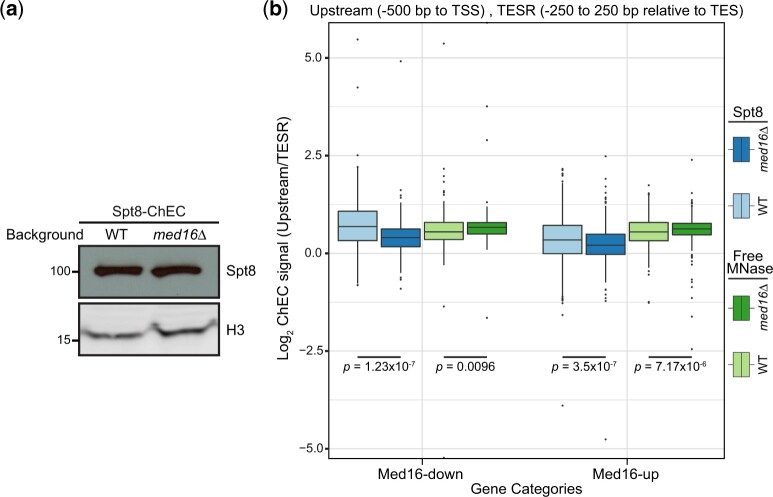
Spt8 occupancy decreases in *med16*Δ. a) Western blot of SAGA subunit Spt8 (Spt8-3xFLAG-MNase) in WT and *med16*Δ cells. b) Boxplots of log_2_ upstream/TESR of SAGA and free MNase ChEC-seq signal from the WT and *med16*Δ strains for Med16-down (*n* = 187) and Med16-up (*n* = 745) genes. Statistical differences between groups were assessed by Wilcoxon rank-sum test.

Although Mediator’s tail and SAGA regulate an overlapping set of genes (Ansari *et al.* 2012), and both SAGA and Mediator bind the UASs/enhancers of almost all genes (Grünberg *et al.* 2016; Baptista *et al.* 2017; Donczew *et al.* 2020), how the Mediator tail and SAGA coregulate their targets is not well understood. Given that previous work has shown that Mediator influences SAGA recruitment to specific genes (Qiu *et al.* 2005; Yarrington *et al.* 2020), we sought to determine if SAGA occupancy is altered upon Med16 loss, which results in cMed/tail separation (Zhang *et al.* 2004; Saleh *et al.* 2021). To that end, we used chromatin endogenous cleavage and high-throughput sequencing (ChEC-seq) (Zentner *et al.* 2015) to investigate the effect of Med16 depletion on the occupancy of the SAGA core subunit Spt8 in both wild-type (WT) and *med16*Δ cells*.* We chose Spt8 because it is a core subunit specific to SAGA that directly contacts TBP (Sermwittayawong and Tan 2006; Papai *et al.* 2020; Wang *et al.* 2020). We focused our analysis on Med16-regulated genes (Med16R genes), which represent genes differentially regulated in the same direction in both stable deletion and conditional depletion conditions of Med16 (Saleh *et al.* 2021), thus limiting the analysis to genes likely to be directly affected by Med16 loss. The level of Spt8 did not change between WT and *med16*Δ cells (Fig. 1a). To allow comparison between ChEC-seq data from Spt8 and other SAGA subunits (described below), we opted to use an internal normalization strategy that we described previously (Saleh *et al.* 2021). Briefly, ChEC-seq signal in the upstream regulatory region, defined as a 500-bp area upstream of the transcription start site (TSS), was normalized to ChEC-seq signal in an identically sized region centered around the transcription end site (TES) in the same replicate/sample. We must note that the TSS and TES annotations for sacCer3 indicate open reading frame start and end sites, respectively. The TES region was chosen for normalization because this region is naturally nucleosome depleted, thus accounting for chromatin accessibility, and is not associated with any PIC assembly or coactivator occupancy (Fan *et al.* 2010; Brogaard *et al.* 2012).

Our results indicate a significant reduction in Spt8 occupancy at both Med16-down and Med16-up genes (Fig. 1b). It should be noted that the enrichment levels of Spt8-MNase were comparable to those of micrococcal nuclease with a nuclear localization signal (free MNase) (Fig. 1b), which is probably due to the low signal-to-noise ratio of Spt8 ChEC-seq. However, the Spt8 ChEC-seq replicates showed good reproducibility (Supplementary Fig. 1a) and the effect observed was consistent across biological replicates (Supplementary Fig. 1b) and contrasted with the change observed with free MNase, which shows a slightly higher signal in *med16*Δ compared to WT cells (Fig. 1b and Supplementary Fig. 1c), consistent with previous reports showing that chromatin from *med16*Δ cells has a higher sensitivity to MNase digestion (Jiang and Stillman 1992; Macatee *et al.* 1997). Furthermore, the average profile of Spt8 ChEC-seq signal around the TSS of Med16R genes confirms the reduction of Spt8 occupancy at Med16-down genes but does not show the reduction observed in Med16-up genes (Supplementary Fig. 1c). Taken together, these data suggest that Spt8 occupancy is reduced at Med16-down genes upon cMed/tail separation.

### cMed/tail separation does not alter Spt3 occupancy at Med16R genes

Our data thus far suggest that Spt8 occupancy is reduced at Med16-down genes in *med16*Δ cells. However, while Spt8 is a core subunit of SAGA, it is not a component of the highly related SAGA-like (SLIK) complex, also known as SAGA-like Spt8 absent (SALSA) (Pray-Grant *et al.* 2002; Sterner *et al.* 2002). SLIK/SALSA is differentiated from SAGA by the absence of the core subunit Spt8 as well as the presence of a C-terminally truncated version of Spt7 (Sterner *et al.* 2002). Given that SAGA can be converted to SLIK/SALSA by the action of the Pep4 peptidase (Spedale *et al.* 2010), we sought to investigate if SLIK/SALSA occupancy differs from that of SAGA. To address this question, we performed Spt3 ChEC-seq in both WT and *med16*Δ cells focusing on Med16R genes to limit the analysis to genes likely to be directly affected by Med16 depletion.

**Fig. 2.**
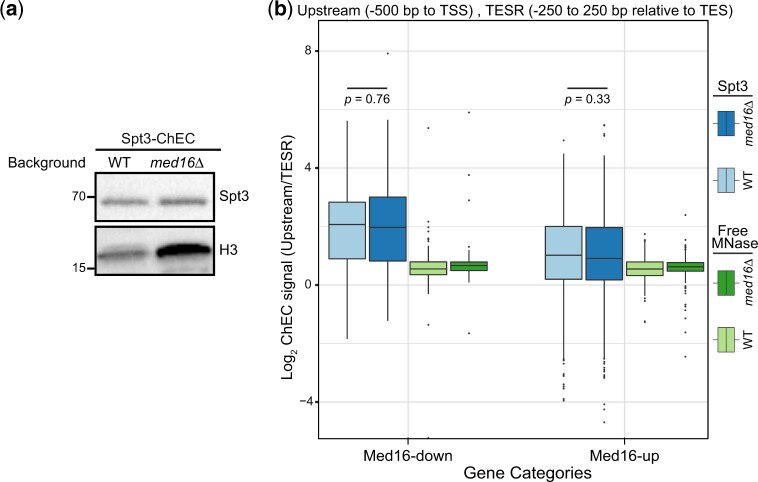
Spt3 occupancy is not changed in *med16*Δ. a) Western blot of SAGA subunit Spt3 (Spt3-3xFLAG-MNase) in WT and *med16*Δ cells*.* b) Boxplots of log_2_ upstream/transcription end site region (TESR) of SAGA and free MNase ChEC-seq signal from the WT and *med16*Δ strains for Med16-down and Med16-up genes. Statistical differences between groups were assessed by Wilcoxon rank-sum test.

The level of Spt3 changes between WT and *med16*Δ cells (Fig. 2a). However, the internal normalization method previously described should control for the difference in protein levels between the two conditions. Our results indicate that there is no significant difference in the normalized Spt3 ChEC-seq signal between WT and *med16*Δ cells (*P > *0.05 by pairwise Wilcoxon rank-sum test) (Fig. 2b)*.* Furthermore, Spt3 ChEC-seq replicates showed good correlation (Supplementary Fig. 2b). However, no consistent changes were observed across three biological replicates (Supplementary Fig. 2a). Taken together, our data suggest that the occupancy of SAGA but not SLIK/SALSA is altered at Med16R genes upon cMed/tail separation.

### SAGA participates in Med16-depletion-dependent transcriptional overactivation

Our data thus far show that Spt8 but not Spt3 occupancy at SAGA target genes is dependent on the cMed/tail connection. Recruitment of SAGA to the genome by TFs is mainly mediated by the Tra1 subunit (Brown *et al.* 2001); thus, it stands to reason that its recruitment is generally dependent on Tra1. However, Tra1 is shared with another coactivator complex: nucleosome acetyl transferase of H4 (NuA4) (Doyon and Côté 2004). Thus, depleting Tra1 to study the effect of impairing SAGA recruitment to the genome by Tra1 removal would result in confounded results. However, removal of SAGA’s core subunit Spt20 destabilizes the SAGA complex and results in the separation of the core, Dub, and Tra1 modules (Lee *et al.* 2011). In order to test the effects of SAGA disruption on the transcriptional dysregulation caused by cMed/tail separation, we performed conditional depletion of Spt20 by the auxin-induced degradation (AID) system (Donczew *et al.* 2020). To confirm the destabilizing effect of Spt20 depletion on SAGA, we mapped the binding of Spt3 to the genome by ChEC-seq. Depleting Spt20 did not affect the stability of Spt3 (Supplementary Fig. 3a). As expected, Spt3 occupancy was reduced genome wide upon Spt20 depletion regardless of the gene categories (Supplementary Fig. 3b).

**Fig. 3.**
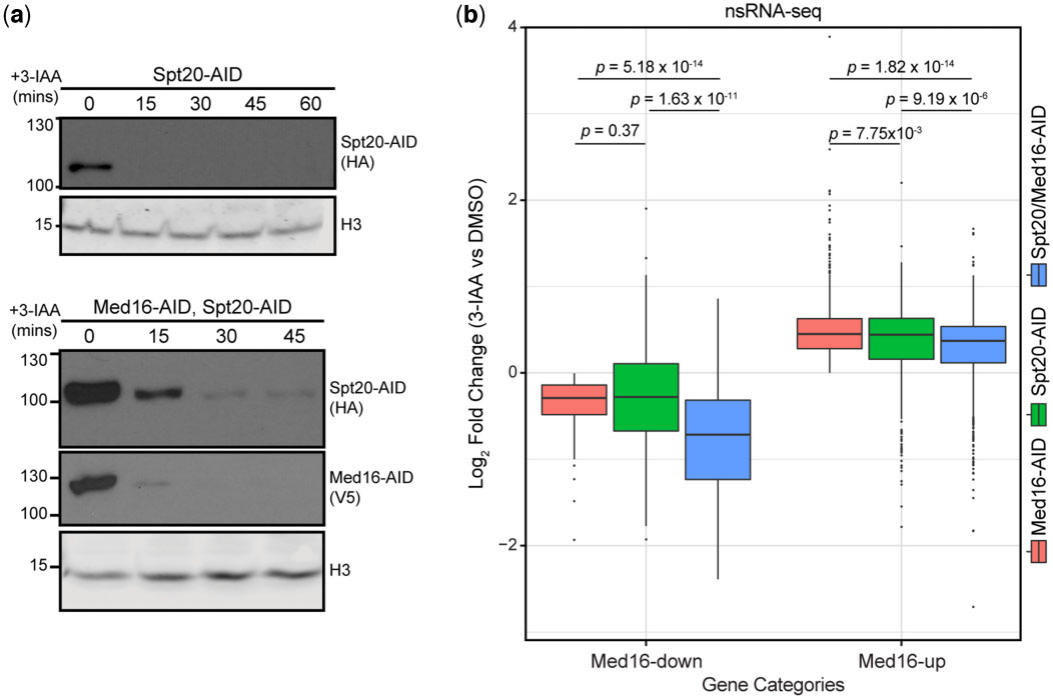
Spt20 contributes to Med16-depletion-dependent transcription overactivation. a) Western blot showing the kinetics of depletion upon addition of 3-IAA to Spt20-AID (single degron) and Med16/Spt20-AID (double degron) upon 3-IAA treatment. b) Boxplots of log_2_ fold changes in nsRNA levels of transcripts produced from Med16-AID downregulated and upregulated genes for the Med16-AID, Spt20-AID, and Spt20/Med16-AID 3-IAA versus DMSO comparisons. Statistical differences between groups were assessed by pairwise Wilcoxon rank-sum test with Holm correction for multiple testing

We then wanted to assess the effect of depleting Spt20 on RNAPII transcription, particularly how it modulates the effect of cMed/tail separation on the transcriptome. To that end, we performed newly synthesized RNA-seq (nsRNA-seq) following depletion of Spt20 alone or in combination with Med16 (Fig. 3a) in biological triplicates (Supplementary Fig. 4a). Spt20 depletion downregulated Med16-down genes similarly to Med16-AID (*P = *0.37 by pairwise Wilcoxon rank-sum test) (Fig. 3b). Concurrent depletion of Spt20 and Med16 reduced the expression of Med16-down genes to a significantly greater extent than either Med16-AID (*P *=* *5.18 × 10^−14^ by pairwise Wilcoxon rank-sum test) or Spt20-AID (*P *=* *1.63 × 10^−11^ by pairwise Wilcoxon rank-sum test) alone, indicating cooperativity between tailed Mediator and SAGA at these genes, consistent with the enrichment of CR genes in Med16-down genes (Saleh *et al.* 2021). Notably, Spt20 depletion alone increased the expression of Med16-up genes, though to a significantly lesser extent than Med16 alone (*P *=* *7.75 × 10^−3^ by pairwise Wilcoxon rank-sum test) (Fig. 3b). Concurrent depletion of Spt20 and Med16 attenuated the upregulation of Med16-up genes compared to Med16-AID (*P *=* *1.82 × 10^−14^ by Wilcoxon rank-sum test) (Fig. 3b), indicating a role for SAGA integrity in Med16-depletion-dependent transcriptional upregulation.

Given that Spt20 depletion resulted in moderate overactivation of Med16-up genes and these genes are enriched in TFIID-dependent genes (Saleh *et al.* 2021), we asked if Spt20 depletion has a similar effect on the entirety of TFIID regulated genes as well as all CR genes. Interestingly, Spt20 depletion resulted in stronger upregulation of TFIID-dependent genes compared to Med16-AID (Supplementary Fig. 4b), and this upregulation is dependent on the presence of Med16. On the other hand, CR genes were more downregulated on average in Spt20-AID compared to Med16-AID. Moreover, Med16/Spt20-AID exhibited a stronger downregulation of CR genes compared to both single degrons (Supplementary Fig. 4). Taken together, these data suggest that tailed Mediator and SAGA cooperate in regulating an overlapping set of genes consistent with previous reports (Ansari *et al.* 2012), and that SAGA has a role in Med16-depletion-dependent transcriptional upregulation.

### TFIID subunits have altered occupancy at Med16-down genes

**Fig. 4.**
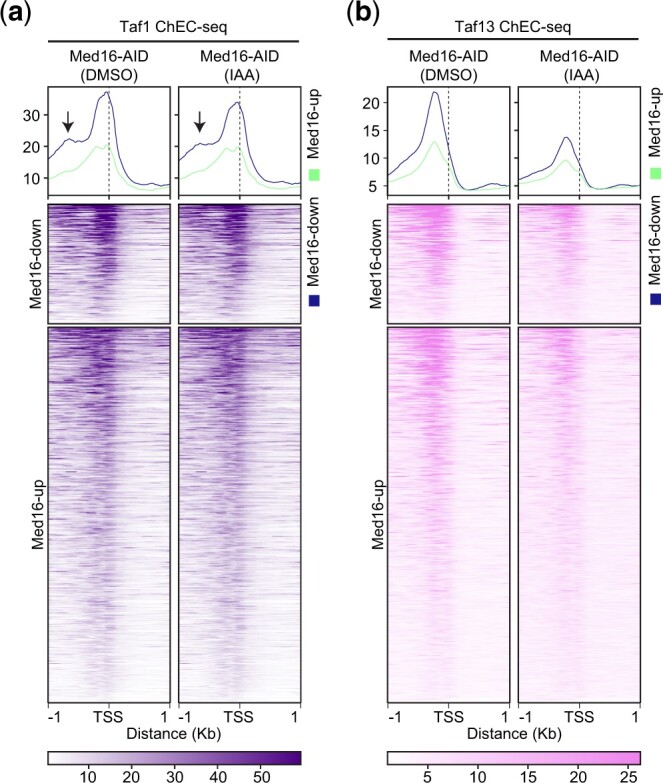
TFIID subunits ChEC-seq. a) Average plots and Heatmaps of Taf1 ChEC-seq signal at 2 Kb region centered around the TSS of Med16-up and Med16-down genes, dashed line indicates TSSs, and arrows indicate “shoulder.” b) Same as (a) but for Taf13 ChEC-seq.

Given the importance of TFIID for all RNAPII transcription and the enrichment of TFIID-dependent genes in Med16-up genes (Saleh *et al.* 2021), we asked if Med16 depletion has any effect on TFIID occupancy genome-wide. To address this question, we performed ChEC-seq on the TFIID-specific Tafs, Taf1 and Taf13. Both Taf1 and Taf13 contact TBP and reside in separate lobes of TFIID, with Taf1 residing in lobe A (Taf1 lobe) and Taf13 residing in lobe C (twin lobe) (Kolesnikova *et al.* 2018). Taf1 and Taf13 protein levels were not affected by Med16 degradation (Supplementary Fig. 5, a and b), and ChEC-seq replicates showed high correlation between the 2 biological replicates of each condition (Supplementary Fig. 5, c and d). We again focused our analysis on Med16R genes since these are the genes likely to be directly affected by Med16 depletion. Taf1 ChEC-seq exhibited an average profile characterized by a peak apex located closer to the TSS compared to the Taf13 profile at both Med16-up and Med16-down genes (compare Fig. 4a and Fig. 4b). The Taf1 average profile at Med16-down genes also showed 2 peaks upstream of the TSS (Fig. 4a). However, the Taf13 average profiles showed a smaller “shoulder” compared to that observed in the Taf1 profile at ∼500-bp upstream of the TSS of Med16-down genes (see arrow in Fig. 4a). Taf13 showed a strong reduction, compared to Taf1, in ChEC-seq signal at both Med16-up and Med16-down TSSs upon Med16 depletion (Fig. 4b). However, we did not detect any changes in the profile shapes of both Taf1 and Taf13 at Med16R genes following Med16 depletion. Taken together, these data suggest that Med16 depletion alters the binding of the TFIID subunit Taf13 at both Med16-up and Med16-down genes.

### TFIID is essential for Med16-depletion-dependent transcriptional overactivation

**Fig. 5.**
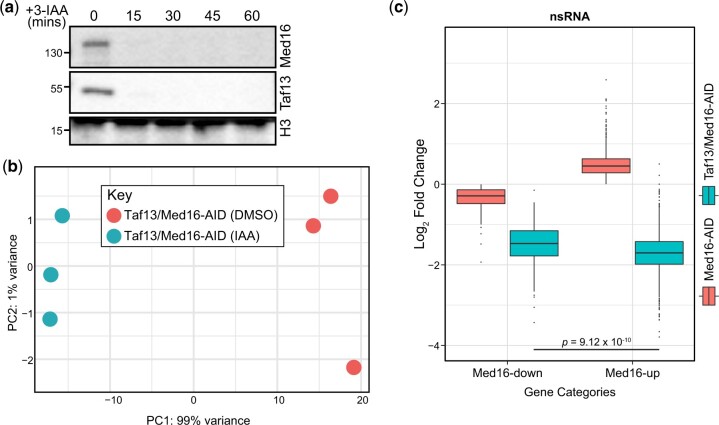
Taf13 is required for Med16-depletion-dependent transcription overactivation. a) Western blotting showing the kinetics of depletion upon the addition of 3-IAA to cells with Taf13/Med16-AID. b) PCA plot of the biological replicates of 3-IAA and DMSO treated Taf13/Med16-AID cells. c) Boxplots of log_2_ fold changes in nsRNA levels of transcripts produced from Med16-down and Med16-up genes for the Med16-AID and Taf13/Med16-AID 3-IAA versus DMSO comparisons. Statistical differences between groups were assessed by pairwise Wilcoxon rank-sum test.

Given that Med16 depletion alters the binding of TFIID subunit Taf13 at Med16R genes, we wondered if TFIID function is required for the transcriptomic changes observed in Med16-depletion. To that end, we performed nsRNA-seq in a strain where both Med16 and Taf13 were targeted by AID (Fig. 5a) using biological triplicates (Fig. 5b). Acute depletion of Taf13 is known to result in global transcriptional downregulation (Donczew *et al.* 2020). Similarly, the combined depletion of Med16 and Taf13 did not deviate from this global downregulation (Fig. 5c). Consistent with the enrichment of TFIID-dependent genes among Med16-up genes (Saleh *et al.* 2021), Med16/Taf13-AID showed a stronger downregulation of Med16-up genes compared to Med16-down genes (*P* = 9.12 × 10^−10^) (Fig. 5c). Taken together, these data show that TFIID is required for the transcriptional overactivation associated with Med16 depletion.

## Discussion

Here, we have investigated the role of the SAGA and TFIID coactivator complexes in transcriptional dysregulation induced by cMed/tail separation. Our results suggest that the transcriptional upregulation observed upon Med16 depletion is dependent on TFIID and, to a lesser extent, SAGA. However, these transcriptional changes do not seem to be mediated by alterations in the recruitment of these complexes to Med16R genes, suggesting that their functions are modulated postrecruitment.

In agreement with previous reports, we found that SAGA occupancy, as determined by Spt3 ChEC-seq, at Med16R genes is not dependent on Mediator’s structural integrity (Leroy *et al.* 2006; Donczew and Hahn 2021). This finding further supports a model where Mediator influences SAGA’s function postrecruitment and not necessarily by altering its recruitment. Indeed, we detected a reduction of Spt8 at Med16-down genes in Med16 deletion; however, whether this reduction is due to differential binding of SLIK/SALSA vs SAGA requires additional evidence. We propose several models to explain our observation of reduced Spt8 at genes downregulated upon Med16 deletion (see below). Spt8 attachment to SAGA depends on the presence of full length Spt7 (Pray-Grant *et al.* 2002; Sterner *et al.* 2002; Papai *et al.* 2020; Wang *et al.* 2020). Spt7 C-terminus is cleaved posttranslationally by the action the peptidase Pep4 (Spedale *et al.* 2010). This cleavage results in loss of Spt8 connection to the rest of SAGA complex (Sterner *et al.* 2002). It is not clear if this cleavage occurs before, after, or independent of SAGA recruitment to target genes. Furthermore, little is known about what triggers Spt7 cleavage and thus, the release of Spt8 from SAGA. However, Spt8 was shown to have a role in promoting the activating functions of TFIIA N-terminal (Warfield *et al.* 2004). Given these present observations, we propose 3 nonmutually exclusive explanations for these observations. The first model is that tailed Mediator blocks Spt7 cleavage at Med16-down genes until the PIC is properly assembled; and in the case of Med16 depletion, reduced cMed recruitment at Med16-down genes results in less PIC assembly and therefore, more Spt7 cleavage and subsequently less Spt8 associated with SAGA and hence lower signal at genes. This model is supported by the negative genetic interactions between multiple tail subunits and Spt8 (Collins *et al.* 2007). Furthermore, the release of TBP from SAGA is dependent on TFIIA’s interaction with SAGA (Papai *et al.* 2020), which is mediated by Spt8 (Warfield *et al.* 2004). However, it is not known if the presence of an assembled PIC in proximity to SAGA can block Spt7 cleavage. The second model proposes that the tailed Mediator promotes posttranslational modification of Spt7 or Spt8, which triggers Spt7 cleavage by Pep4 and the subsequent release of Spt8 from SAGA. The assumptions of this model, posttranslational phosphorylation of Spt8 residues, are supported by the presence of multiple phosphorylated serine residues in Spt8 (Chi *et al.* 2007; Albuquerque *et al.* 2008); however, the nature of the kinase responsible and the consequences of this posttranslational modification is still to be determined. The third model is that cMed/tail separation results in conformational changes in SAGA at Med16-down genes that results in an apparent reduction in Spt8 occupancy due to movement of the C-terminal MNase tag away from DNA.

Another interesting finding that was revealed in this study is the reduction of Taf13 ChEC-seq signal at Med16R genes. One can argue that this is not necessarily a reduction in TFIID occupancy because Taf1 did not show a similar decrease. A reasonable explanation for Taf13 signal reduction upon Med16 depletion may be a change in the conformation rather than level of bound TFIID. Indeed, recent complete structures of TFIID-containing PIC on several promoters have shown large conformational changes in TFIID upon binding promoters (Patel *et al.* 2018; Chen *et al.* 2021). Indeed, similar structural rearrangements were proposed for yeast TFIID, and these conformational changes seem to play a role in facilitating reinitiation (Joo *et al.* 2017). Thus, it remains to be tested if Med16-depletion results in similar changes in the conformation of promoter-bound TFIID.

Taken together, our data suggest that the connection of the core and tail Mediator modules alters the function of other coactivator complexes after their recruitment to their targets. However, the exact mechanism by which Mediator exerts this effect remains to be tested.

## Supplementary Material

jkac290_Supplementary_Figure_Legends

jkac290_Supplementary_Figure_S1

jkac290_Supplementary_Figure_S2

jkac290_Supplementary_Figure_S3

jkac290_Supplementary_Figure_S4

jkac290_Supplementary_Figure_S5

jkac290_Supplementary_Table_S1

jkac290_Supplementary_Table_S2

## Data Availability

All datasets generated in this work have been deposited in GEO (GSE207371). Supplemental material is available at G3 online.
