# Supplementary material for: Involvement of the SAGA and TFIID coactivator complexes in transcriptional dysregulation caused by the separation of core and tail Mediator modules
Source: G3 (Bethesda). 2022 Nov 4;12(12):jkac290. doi: 10.1093/g3journal/jkac290 (PMC9713439; doi:10.1093/g3journal/jkac290)
Supplement: jkac290_Supplementary_Figure_Legends [file jkac290_supplementary_figure_legends.docx]

**Figure S1 –** (A) Correlation matrix of Spt8 ChEC-seq replicates in WT and *med16Δ* cells (-1 kb to +1 kb relative to the TSSs of all genes). (B) Boxplots of log_2_ upstream/TESR of Spt8 ChEC-seq biological replciates from the WT and *med16*Δ strains for Med16-down and Med16-up genes. (C) Average plot and Heatmap of Spt8 ChEC-seq in 2 Kb region centered around the TSS of Med16-up and Med16-down genes. (D) Average plot of Spt8 and Free MNase ChEC-seq in 2 Kb region centered around the TSS of Med16-up (top) and Med16-down (bottom) genes.

**Figure S2 –** (A) Correlation matrix of Spt3 ChEC-seq replicates in WT and *med16Δ* cells (-1 kb to +1 kb relative to the TSSs of all genes). (B) Boxplots of log_2_ upstream/TESR of Spt3 ChEC-seq replicates from the WT and *med16*Δ strains for Med16-down and Med16-up genes. Statistical differences between groups were assessed by Wilcoxon rank-sum test.

**Figure S3 -** (A) Western blot showing the kinetics of depletion and stability of Spt3-3xFLAG-MNase upon treatment by 3-IAA (B) Average plot and Heatmap of Spt3 ChEC-seq in 2 Kb region centered around the TSS of coactivator-redundant genes (CR) (Blue) and TFIID-dependent genes (green).

**Figure S4 -** (A) PCA plot of replicate nsRNA-seq experiments performed in Spt20/Med16-AID and Spt20-AID cells treated with DMSO or 3-IAA. (B) Boxplots of log_2_ fold changes in nsRNA levels of transcripts produced from CR and TFIID genes for the Med16-AID, Spt20-AID, and Spt20/Med16-AID 3-IAA versus DMSO comparisons.

**Figure S5 -** Western blot showing the kinetics of depletion and stability of Taf1-3xFLAG-MNase (A) and Taf13-3xFLAG-MNase (B) upon treatment by 3-IAA. Correlation matrices of ChEC-seq replicates from DMSO- and 3-IAA-treated Med16-AID cells (-1 kb to +1 kb relative to the TSSs of all genes) for (C) Taf1 ChEC-seq and (D) Taf13 ChEC-seq.
