# Supplementary figures and images for: Involvement of the SAGA and TFIID coactivator complexes in transcriptional dysregulation caused by the separation of core and tail Mediator modules
Source: G3 (Bethesda). 2022 Nov 4;12(12):jkac290. doi: 10.1093/g3journal/jkac290 (PMC9713439; doi:10.1093/g3journal/jkac290)

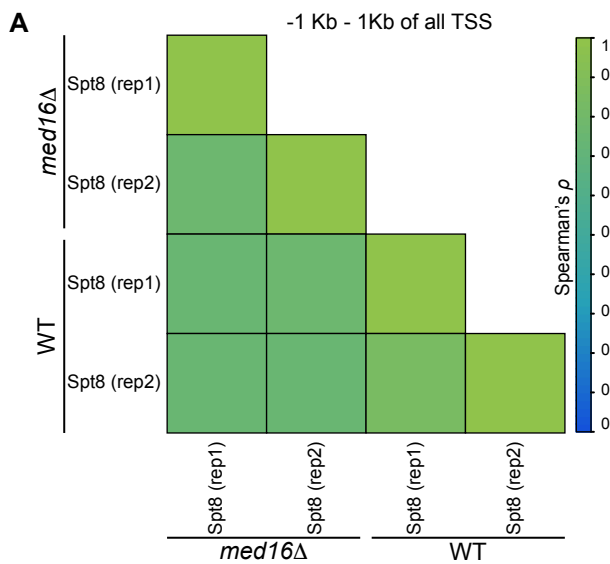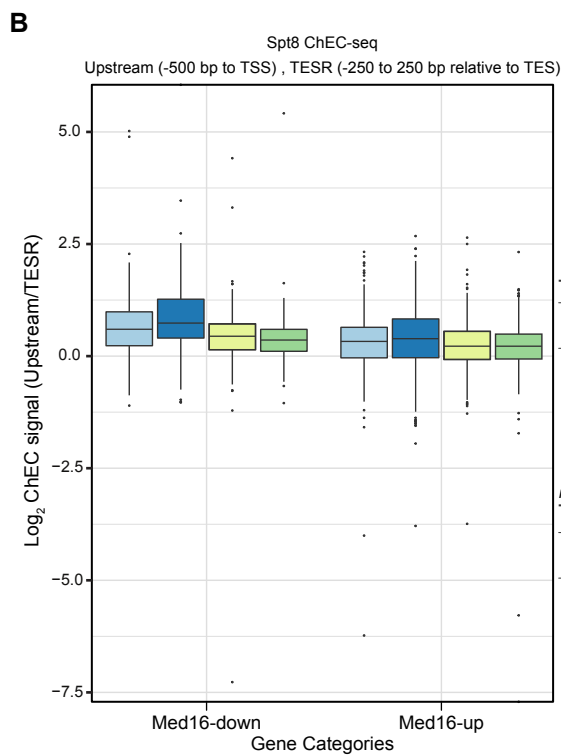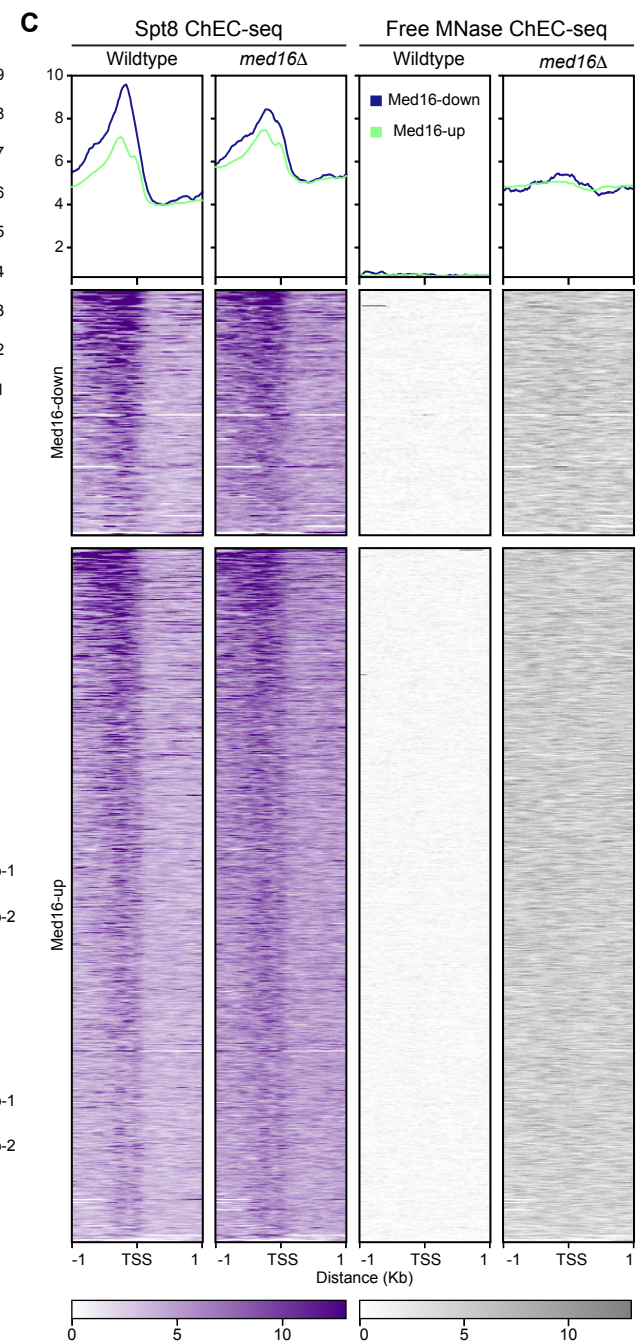

Supplement: jkac290_Supplementary_Figure_S1 [file jkac290_supplementary_figure_s1.pdf]

**A**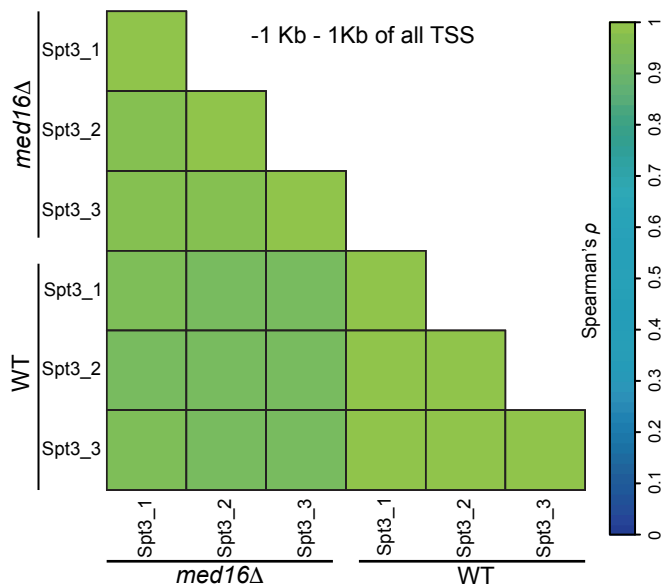**B**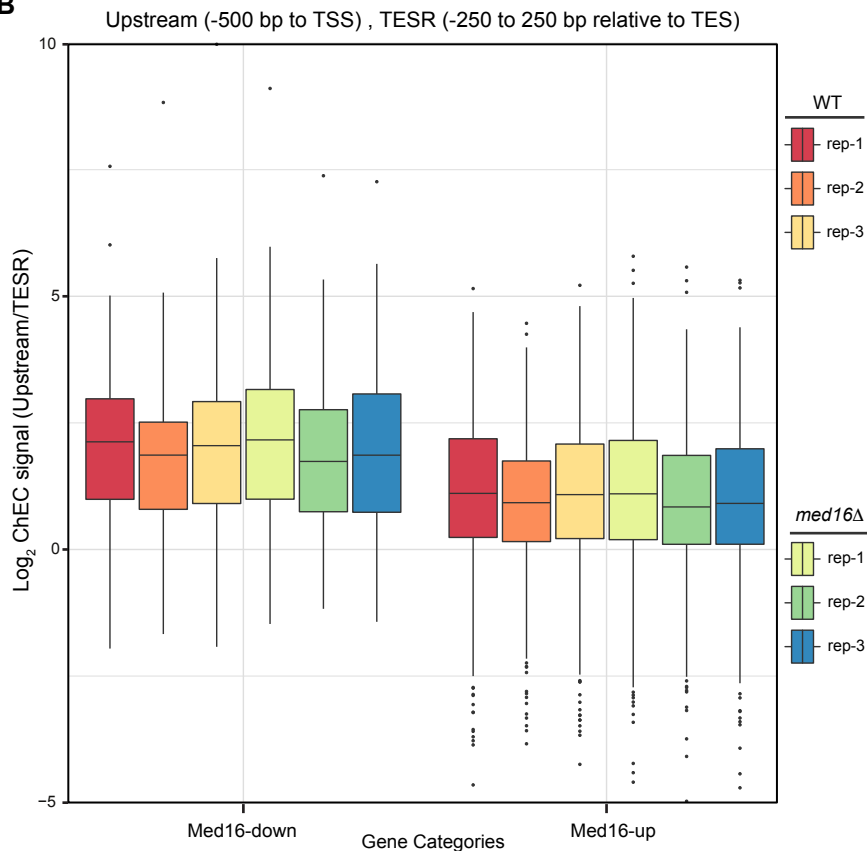

Supplement: jkac290_Supplementary_Figure_S2 [file jkac290_supplementary_figure_s2.pdf]

**A**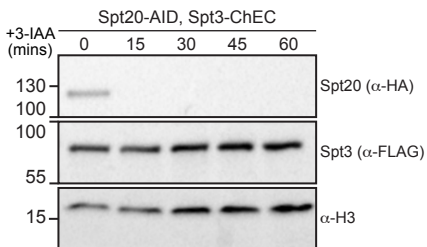**B**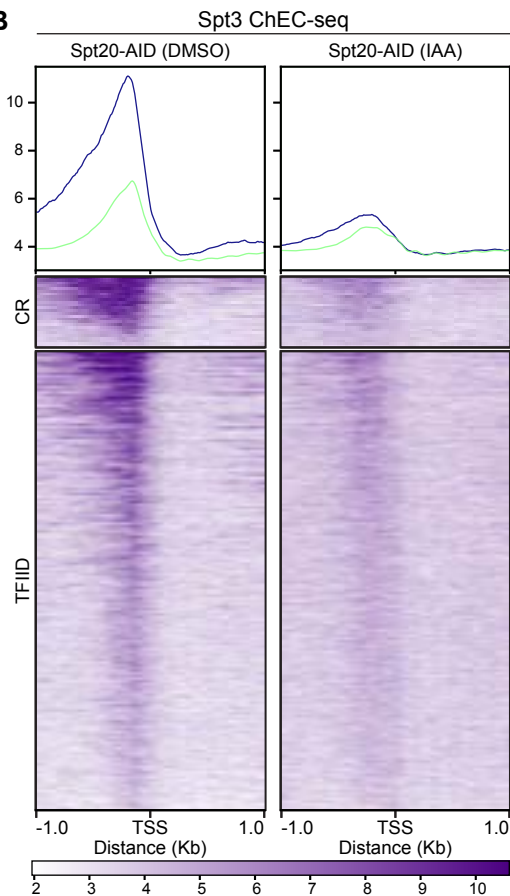

Supplement: jkac290_Supplementary_Figure_S3 [file jkac290_supplementary_figure_s3.pdf]

**A**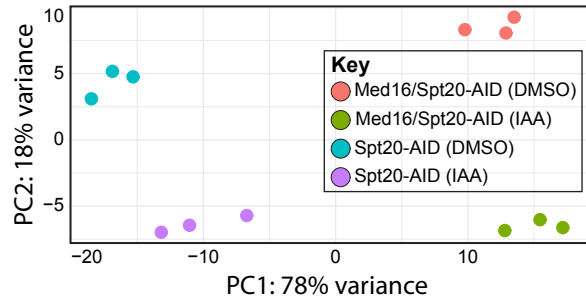**B**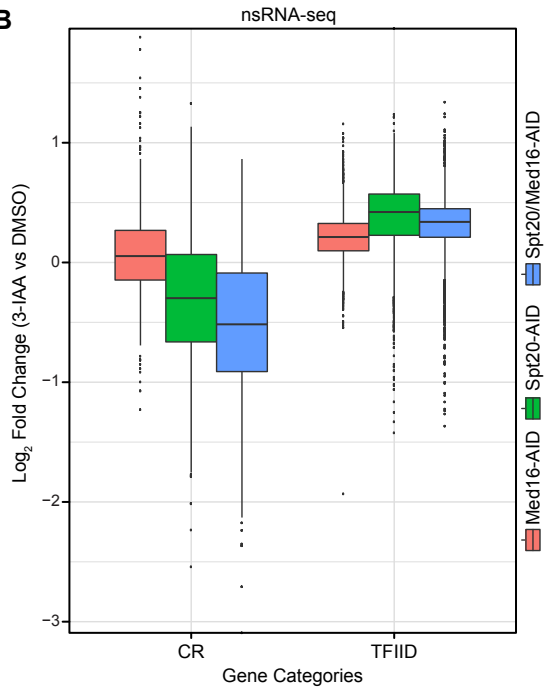

Supplement: jkac290_Supplementary_Figure_S4 [file jkac290_supplementary_figure_s4.pdf]

**A**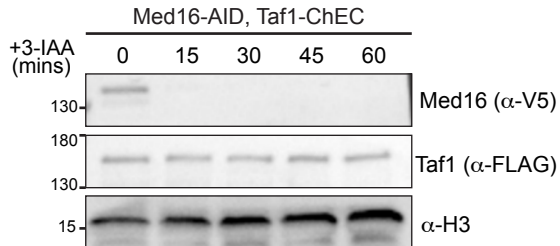**B**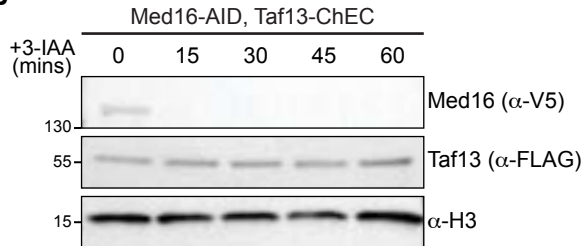**C**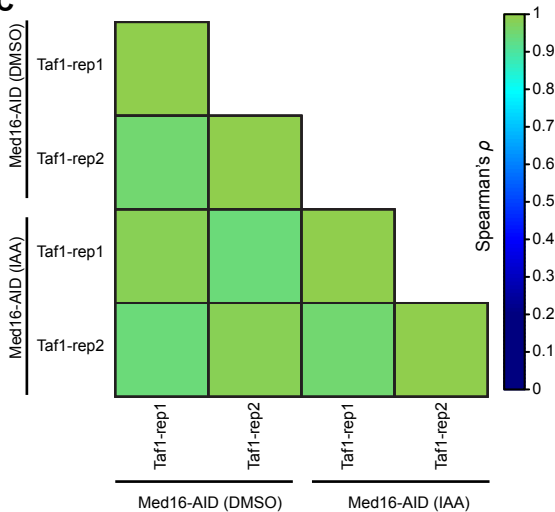**D**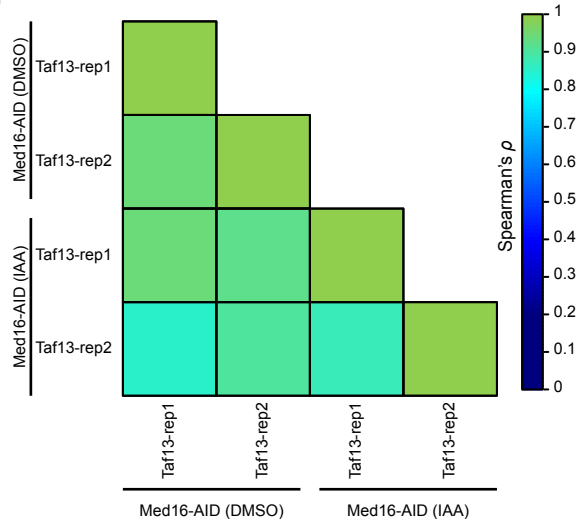

Supplement: jkac290_Supplementary_Figure_S5 [file jkac290_supplementary_figure_s5.pdf]
